# Greater hemodynamic stresses initiate aneurysms on major cerebral arterial bifurcations

**DOI:** 10.3389/fneur.2023.1265484

**Published:** 2023-10-12

**Authors:** Hao Guo, Jian-Feng Liu, Cong-Hui Li, Ji-Wei Wang, Hui Li, Bu-Lang Gao

**Affiliations:** Department of Neurosurgery, The First Hospital, Hebei Medical University, Shijiazhuang, Hebei, China

**Keywords:** computational fluid dynamics, hemodynamic stresses, major cerebral arteries, arterial bifurcation, intracranial aneurysms

## Abstract

**Objective:**

To retrospectively investigate the hemodynamic stresses in initiating aneurysm formation on major cerebral arterial bifurcations with computational fluid dynamics (CFD) analysis.

**Methods:**

The cerebral 3D angiographic data of major cerebral arterial bifurcations of the internal carotid, middle cerebral, anterior cerebral, and basilar arteries in 80 patients harboring bifurcation aneurysms and 80 control subjects with no aneurysms were retrospectively collected for the CFD analysis of hemodynamic stresses associated with aneurysm formation.

**Results:**

Bifurcation angles at major bifurcations in all patients were significantly positively (*P* < 0.001) correlated with the age. At the center of direct flow impingement (CDFI) on the bifurcation wall, total pressure was the highest but dropped rapidly toward the branches. Wall shear stress, dynamic pressure, strain rate, and vorticity were lowest at the CDFI but they increased quickly toward the branches. The bifurcation angle was significantly (*P* < 0.001) enlarged in patients with bifurcation aneurysms than those without them, for all major arterial bifurcations. Most aneurysms leaned toward the smaller arterial branch or the arterial branch that formed a smaller angle with the parent artery, where the hemodynamic stresses increased significantly (*P* < 0.05), compared with those on the contralateral arterial branch forming a larger angle with the parent artery. Following the aneurysm development, all the hemodynamic stresses on the aneurysm dome decreased significantly (*P* < 0.001) compared with those at the initiation site on the bifurcation wall after virtual aneurysm removal. With the decrease of bifurcation angles, all the hemodynamic stresses decreased.

**Conclusion:**

The formation of intracranial aneurysms on major intracranial arterial bifurcations is significantly associated with locally abnormally augmented hemodynamic stresses, which must be reduced.

## Introduction

Cerebral aneurysms are not uniformly distributed in the cerebrovascular tree even though some risk factors, including family history, smoking, and hypertension, exist for aneurysm formation, which impact cerebral arteries as a whole (1). Cerebral aneurysms usually form at arterial bifurcations and at areas of sharp arterial curvatures (2). They also form at certain large arterial bifurcations; for example, those of the middle and anterior cerebral arteries are more frequent locations of aneurysm formation than others (3). The reasons for this are unknown and probably associated with intrinsic local differences, including local hemodynamic stresses, which may contribute to aneurysm formation. Sharp arterial curvatures and bifurcations may cause abnormally augmented hemodynamic forces to start aneurysm formation, and hemodynamic stresses at an arterial bifurcation have been demonstrated to initiate maladaptive arterial remodeling with aneurysmal features in dog experiments (4). Other animal experiments also demonstrated that enhanced hemodynamic forces at the basilar bifurcation apex can activate destructive remodeling and aneurysm development (5, 6). Hemodynamic forces, such as wall shear stress (WSS), pressure, and vorticity, may play a crucial role throughout the aneurysm formation process, including aneurysm activation, progression, and rupture (7). Clinically, cerebral aneurysms tend to occur on big intracranial arterial bifurcations of the internal carotid, middle cerebral, anterior cerebral, and basilar arteries, with aneurysms on the anterior cerebral artery bifurcation (anterior communicating artery aneurysms) accounting for over 25% of all intracranial aneurysms (8). In recent years, computational fluid dynamics (CFD) technology and 3D imaging have developed rapidly, which made CFD analysis of aneurysm development feasible. Nonetheless, earlier studies of intracranial aneurysm hemodynamic analysis mainly centered on blood flow velocity, WSS, and oscillatory shear index (OSI) (9). On vascular bifurcation apexes, where deflected and separated flows and vortexes exist, the WSS, total and dynamic pressure, strain rate, and vorticity have all been augmented compared with those on a straight artery. Based on this, it was hypothesized that only these abnormally enhanced hemodynamic stresses jointly activate aneurysm development on major arterial bifurcation apexes. At present, there are few studies that have investigated these hemodynamic stresses associated with aneurysm formation on big arterial bifurcation apexes with a large dataset of specific 3D imaging of patients with or without intracranial aneurysms. The hemodynamic stresses on big intracranial arterial bifurcation apexes, with a method of virtual aneurysm removal combined with CFD analysis of patients' specific imaging data to reveal the exact site of aneurysm activation caused by hemodynamic forces, were thus studied and presented in this article.

## Materials and methods

This retrospective one-center study was conducted from March 2016 to February 2021 in our hospital, and informed consent was waived by the ethics committee of our hospital because of the study's retrospective design. The datasets of consecutive patients with 3D digital subtraction angiography (DSA) were retrospectively included. All clear 3D imaging data of big vascular bifurcations were included, and patients with unclear imaging datasets were excluded.

The 3D angiographic datasets were used for CFD analysis after reconstruction for surface rendering using the Amira software (v 5.2.2 Visual Imaging, Konrad-Zuse-Zentrum Berlin, Germany). Surface smoothing, virtual removal, and reconstruction of the aneurysm and arteries were conducted using the Meshlab software (Meshlab v1.3.4, Visual Computing Lab, ISTI, CNR) (10). Polyhedral meshes with a high resolution were produced using the Sharc Harpoon software (Sharc, MA, UK) at ~1,000,000 cells per sample. The finite-volume approach was followed using Fluent software (Ansys version 12.0.16, Lebanon, NH, USA); a rigid nonslip wall condition, a blood density of 1,070 kg/m^3^, and a blood viscosity of 3.5 cP (11) were used, with the inlet rate set at 0.1 m/s and the pressure for the outlet set at 0. Because preliminary CFD analysis performed at the peak systole using time-dependent runs in the cardiac cycle for five cases showed similar results to that of the steady-state analyses, the steady-state approach was chosen for CFD analysis to minimize variability. Ensight software (Version 9.0; CEI, Apex, NC, USA) was used for the post-processing of the CFD data.

**Figure 1:**
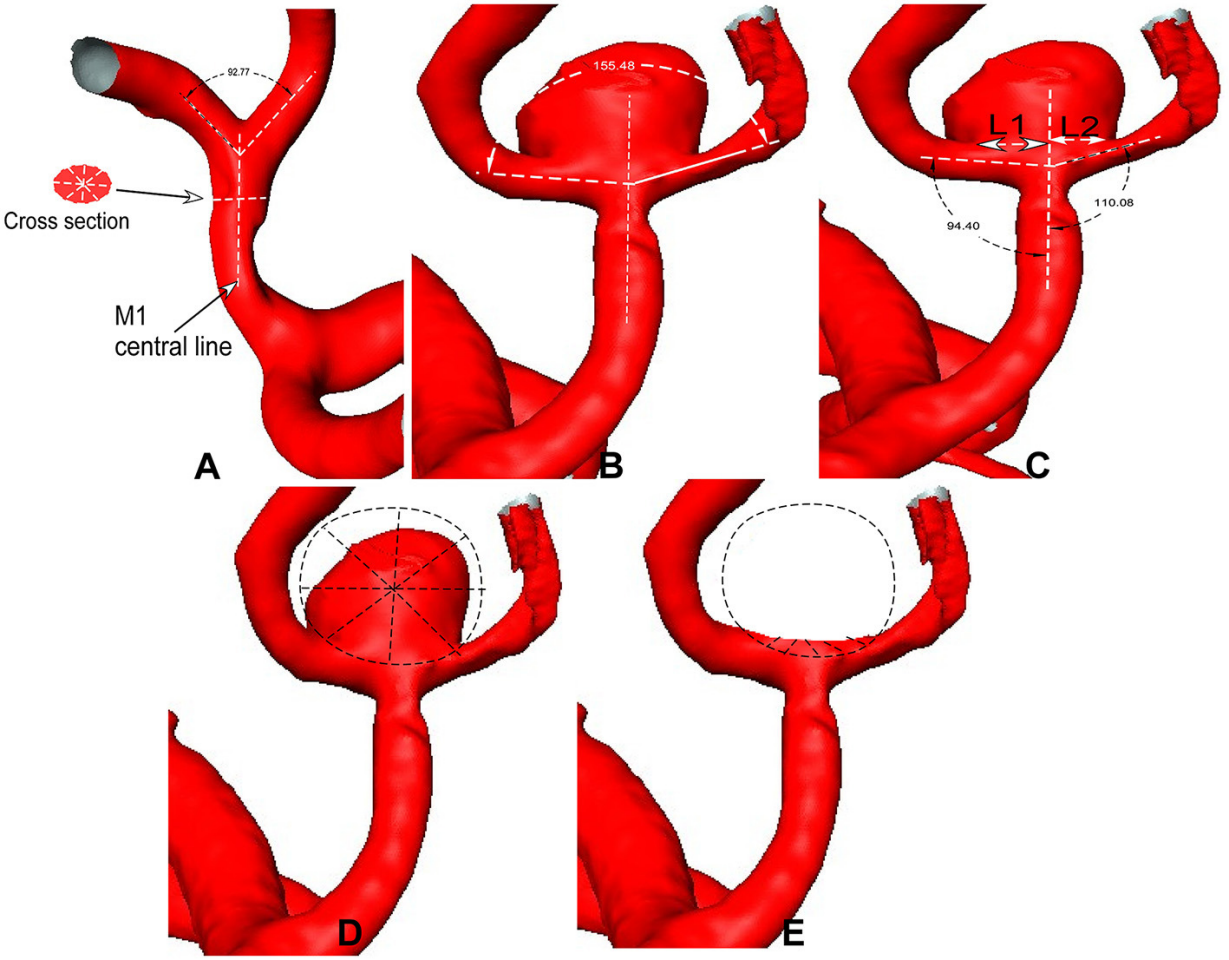
**(A)** Middle cerebral artery (MCA) bifurcation is used to show the measurement. **(A)** The bifurcation angle is measured between two M2 segments (bifurcation angle = 92°), and the M1 diameter is measured in a patient without aneurysms. **(B)** The bifurcation angle is measured in the MCA bifurcation with an aneurysm (bifurcation angle = 155°). **(C)** The bidirectional arrows are used for the aneurysm neck length (L1 and L2), which is divided by the midway line of the parent artery. The angles between M1 and M2 segments are measured. **(D, E)** Sphere sampling is performed on the aneurysm dome **(D)** and at the aneurysm-triggering location after virtual aneurysm removal **(E)**.

The parent artery diameter was measured at the midway point between the bifurcation apex and the last branching point on the parent artery (12). The daughter branch diameter was measured at a distance of 5 mm beyond the bifurcation apex. For the anterior cerebral artery (ACA) bifurcation, the midway point of the anterior communicating artery (Acom) was measured because the Acom was short. The diameter was measured four times before calculating an average value for analysis (Figure 1A). The angle formed between the branch and the parent artery was measured. Three dots were used to measure the arterial bifurcation angle by placing the central point at the bifurcation tip consistent with the parent artery's central axis, while the other two dots marked the proximal segments of the two branch arteries. A similar approach was followed to measure the angle between the parent and branch arteries (Figure 1). Aneurysm deviation was assessed based on the intersection of the aneurysm neck with the parent artery's central line (Figure 1C), and the aneurysm neck was divided into two sections: L1 and L2. The deviation was determined according to the longer sections of L1 and L2. The aneurysm deviated toward the L1 side if L1 > L2 and toward the L2 side if L1 < L2.

**Figure 2:**
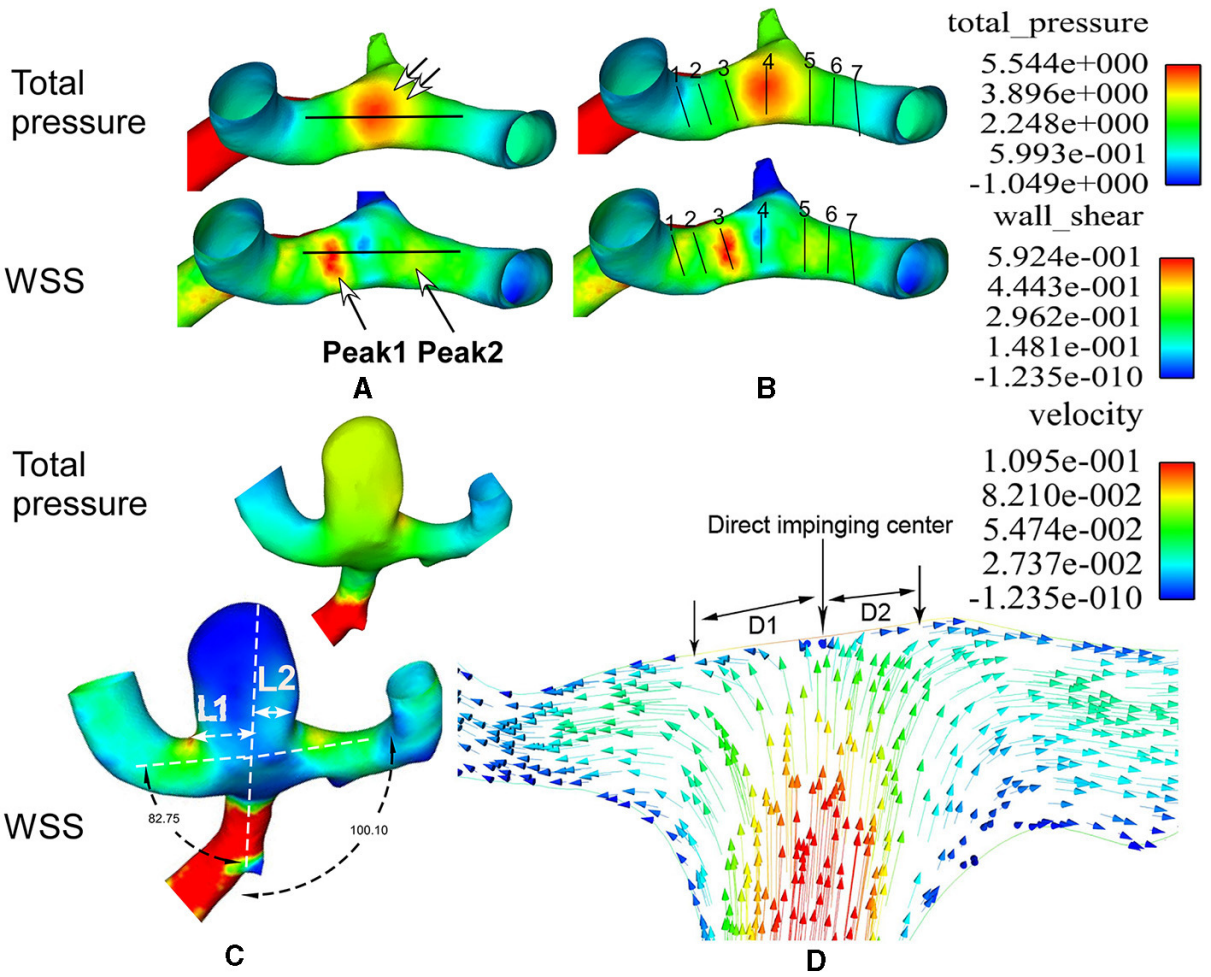
Lines are used to sample the hemodynamic stresses and flow direction at the middle cerebral artery (MCA) bifurcation. **(A, B)** One longitudinal line **(A)** and seven transverse lines **(B)** are used on the bifurcation wall to sample the hemodynamic parameters. The longitudinal line **(A)** crosses the flow direct impingement center (double arrows in **A**). Two wall shear stress (WSS) peaks are demonstrated using two big arrows in **A**. Transverse line 4 is localized at the direct flow impingement center, line 3 at Peak 1, line 5 at Peak 2, and lines 1 and 7 on two MCA bifurcation branches. **(C)** The aneurysm is deviated to the narrower lateral angle (82.75°). L1 and L2 stand for the aneurysm neck length divided by the parent vessel's central line. Because L1 is longer than L2, the aneurysm is deviated to the L1 side. The total pressure on the aneurysm dome is greater than that on the surrounding arteries, while the WSS on the aneurysm dome is decreased compared with that on the surrounding arteries. **(D)** The flow velocity is demonstrated at the direct impingement center on the MCA bifurcation wall. The red arrows represent increased flow speed, while the blue arrows represent decreased flow speed. D1 and D2 indicate the distance from the direct flow impingement center (perpendicular to the bifurcation wall) to the location where the flow becomes laminar (parallel to the arterial branch). D1 is on the side of the smaller lateral angle, while D2 is on the side of the larger lateral angle. D1 is longer than D2.

**Figure 3:**
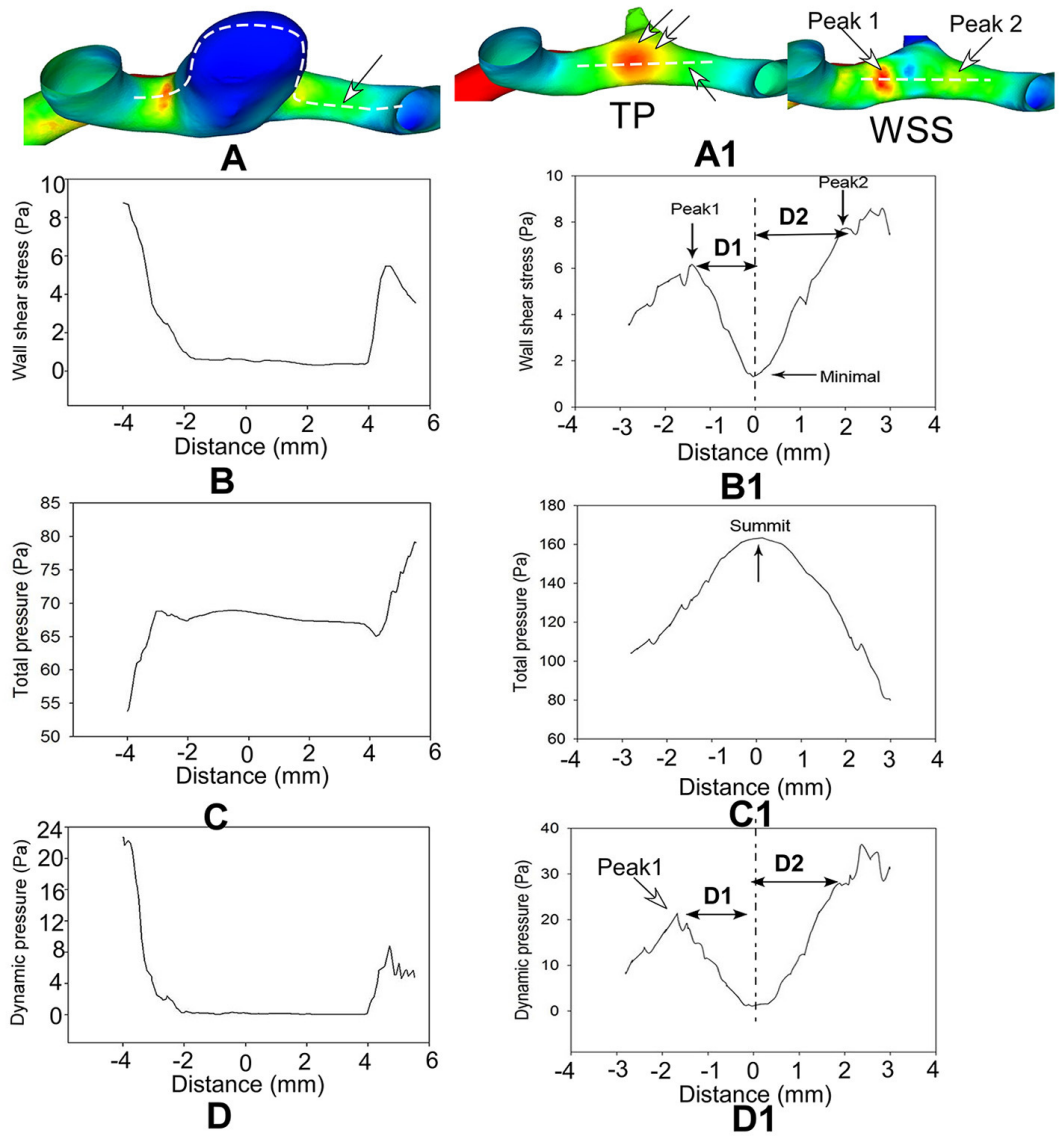
Hemodynamic stresses are analyzed on the longitudinal line across the aneurysm dome (**A–D**, left column) and direct flow impinging center following virtual aneurysm removal (**A1–D1**, right column) in a patient with an anterior communicating artery aneurysm. Distance 0 stands for the direct flow impingement center (double arrows in TP of **A1** after aneurysm removal **(A1–D1)**]. Two hemodynamic stress peaks (arrows in WSS of **A1**) bestriding the flow direct impingement center on the bifurcation wall following virtual aneurysm removal **(B1, D1)**. The hemodynamic stresses are significantly reduced on the aneurysm dome **(A–D)** but are very high on the bifurcation wall after aneurysm removal **(A1–D1)**. **(D)** (D1 and D2) in **(B1, D1)** represents the distance from the peak value to the minimal value at the direct flow impingement center (0 on the *X*-axis in **B1, D1**). TP, total pressure; WSS, wall shear stress.

When comparing the hemodynamic stresses before and after aneurysm development, the virtual aneurysm removal technique, similar to the approach used by Gao et al. (10) and Mantha et al. (13), was applied to remove the aneurysm for simulating the vessel bifurcation wall before aneurysm development. The hemodynamic stresses on the aneurysm dome and initiation site were sampled by employing a sphere sampling technique (Figure 1D). To sample the hemodynamic stresses on the bifurcation wall, a longitudinal line was drawn on the bifurcation wall before aneurysm initiation (Figure 2A). Additionally, seven crossing (or transverse) lines that were perpendicular to the longitudinal lines were also drawn (Figure 2B). Transverse line 4 was across the direct flow impinging center, lines 1–3 were on one arterial branch, and lines 5–7 were toward the other arterial branch (Figure 2C). Lines 3 and 5 were across the center of the two WSS peaks (red areas in the wall shear stress in Figure 2B) beside the direct flow impinging zone. A longitudinal line was also made through the aneurysm dome (Figure 3A) or the direct flow impinging center with aneurysm removal (Figure 3B) for sampling hemodynamic stresses. After the virtual aneurysm removal, the profiles of vorticity, WSS, dynamic pressure, and strain rate on the longitudinal line exhibited two peaks (right column in Figure 3B), and Peak 1 was defined as the peak location on the arterial branch that formed a smaller angle with the parent vessel, while Peak 2 was defined as the peak location on the arterial branch that formed a larger angle with the parent vessel. Transverse line 3 was right localized at Peak 1, while transverse line 5 was right localized at Peak 2.

### Statistical analysis

The statistical analysis was conducted using JMP software (Version 10.0.2, SAS Institute, Cary, NC, USA). The correlation of parameters was assessed by multivariable analyses using the least squares linear regression approach, and the comparison between different groups was made by conducting Student's *t*-test after confirming the normal distribution of the data. The statistical significance was defined as *P* < 0.05.

## Results

### Subjects

Eighty patients with intracranial aneurysms on big arterial bifurcations, including the ACA (*n* = 20), middle cerebral artery (MCA, *n* = 20), internal carotid artery (ICA, *n* = 20), and basilar artery (BA, *n* = 20) bifurcations, were randomly selected for CFD analysis, with 43 women and 37 men aged 26–92 years (mean 55.8 ± 10.6). Twenty aneurysms were randomly selected for each major arterial bifurcation. The patients exhibited clinical symptoms, including subarachnoid hemorrhage, double vision, headache, face numbness, confusion, and other non-specific symptoms (such as limb weakness, dizziness, and visual impairment). Twenty subjects without cerebral arterial stenosis or intracranial aneurysms were randomly recruited into the control group for each big intracranial arterial bifurcation for comparison with the aneurysm group, including 39 women and 41 men aged 22–81 years (mean 52.2 ± 9.1). The patients' gender, age, aneurysm status, and clinical symptoms were collected. No significant (*P* > 0.05) differences were found in the mean age or gender component between the aneurysm and control groups.

### Arterial bifurcation angle

The bifurcation angles formed between two arterial branches at major intracranial arterial bifurcations were determined in 80 patients with aneurysms and 80 patients without aneurysms. In all patients, the bifurcation angle of all four major arteries was significantly (*R* = 0.26, *P* < 0.001) positively correlated with age. The mean bifurcation angle was significantly (*P* < 0.001) greater in patients with than in those without bifurcation aneurysms (145.6° ± 25.8° for aneurysmal but 131.7° ± 15.7° for non-aneurysmal ICA bifurcation, 148.7° ± 33.2° for aneurysmal but 106.6° ± 25.8° for non-aneurysmal MCA bifurcation, 145.2° ± 6.1° for aneurysmal and 106.1° ± 4.2° for non-aneurysmal ACA bifurcation, and 141.4° ± 12.8° for aneurysmal but 105.5° ± 18.8° for non-aneurysmal BA bifurcation). The lateral angle formed between the parent and either branching artery was significantly (*P* < 0.05) smaller in bifurcations with aneurysms than in bifurcations without aneurysms. Of the 80 bifurcation aneurysms, 68 (85%) aneurysms were deviated toward the branching artery, forming a smaller angle with the parent artery (Figures 1, 2), and 60 (75%) aneurysms were deviated toward the smaller-diameter arterial branch.

### Hemodynamic stresses

Analysis of the hemodynamic forces on the aneurysm dome and initiation site after virtual aneurysm removal (Figure 1D; Table 1) demonstrated that the total pressure, WSS, dynamic pressure, vorticity, and strain rate significantly dropped (*P* < 0.001) on the aneurysm dome compared with those on the aneurysm initiation site (126.2 ± 0.3 vs. 141.0 ± 1.1 Pa for total pressure, 3.0 ± 0.1 vs. 19.3 ± 0.8 Pa for dynamic pressure, 1,243.1 ± 20.5 vs. 3,202.4 ± 89.4 1/S for vorticity, 1.9 ± 0.03 vs. 6.2 ± 0.1 Pa for WSS, and 1,325.42 ± 20.50 vs. 3,330.4 ± 98.8 1/S for strain rate), implying that aneurysm formation was to relieve the energy effect on the arterial bifurcation wall at the aneurysm initiation location.

**Table 1:** Hemodynamic stresses on the aneurysm dome and at the aneurysm initiation site after virtual aneurysm removal (Figure 1D).

	**Aneurysm dome**	**Aneurysm initiation site**
Dynamic pressure (Pa)	10.50 ± 0.31^**^ (0.04–122.49)	133.67 ± 7.20 (91.5–168.9)
Total pressure (Pa)	142.41 ± 0.57^**^ (90.57–219.26)	400.80 ± 5.27 (338.4–417.8)
Vorticity (1/S)	2,315.55 ± 28.56^**^ (245.43–10,507.4)	7,068.67 ± 297.66 (5,478.68–9,366.66)
WSS (Pa)	3.85 ± 0.05^**^ (0.31–16.7)	18.03 ± 0.45 (15.62–22.19)
Strain rate (1/S)	2,412.44 ± 28.38^**^ (321.58–10,112.2)	7,792.88 ± 267.34 (6,354.73–9,787.43)

^**^P < 0.001, significant compared with those on the aneurysm initiation site after virtual aneurysm removal.

In the longitudinal line through the aneurysm dome or the direct flow impinging center following virtual aneurysm removal, all the profiles of the hemodynamic parameters on the aneurysm dome were very low and flat, whereas those on the arterial bifurcation oscillated significantly (Figure 3A). Following virtual aneurysm removal, the total pressure reached the peak, where the vorticity, WSS, dynamic pressure, and strain rate were at their lowest at the center of direct flow impingement. As blood flow was accelerated from the direct impinging center toward the arterial branches, the total pressure dropped quickly, but all the other parameters increased quickly to reach the peak value in the area immediately adjacent to the direct impinging center (Peaks 1 and 2) (Figure 3B). The WSS, vorticity, dynamic pressure, and strain rate at Peak 1 were significantly (*P* < 0.01, *P* < 0.01, *P* < 0.05, and *P* < 0.05, respectively) greater than those at Peak 2 (152.4 ± 18.6 vs. 91.7 ± 12.2 for dynamic pressure, 19.9 ± 1.8 vs. 13.8 ± 1.3 for WSS, 9,869.6 ± 957.9 vs. 7,000.1 ± 683.7 for vorticity, and 9,860.1 ± 963.7 vs. 7,122.8 ± 707.2 for strain rate). Moreover, the distance from Peak 1 to the direct impingement center was significantly greater than that from Peak 2 to the direct impingement center (1.8 ± 0.1 mm vs. 1.2 ± 0.1 mm, ^**^P < 0.01) (Table 2).

**Table 2:** Hemodynamic stresses at two peaks of 20 ACA bifurcations with or without aneurysms (mean ± SD).

	**Dynamic pressure (Pa)**	**Total pressure (Pa)**	**Wall shear stress (Pa)**	**Vorticity (1/S)**	**Strain rate (1/S)**	**D (mm)**
Peak 1	148.4 ± 121.3^**^ (23.6–513.1)	407.5 ± 30.3 (65.0–1,426.3)	18.7 ± 1.4^**^ (3.5–47.2)	9,858.6 ± 892.2^*^ (1,893.7–23,693.5)	9,790.1 ± 620.4^*^ (1,859.9–24,824.3)	1.6 ± 0.7^**^ (0.39–4.8)
Peak 2	89.9 ± 9.2 (9.8–389.3)	469.3 ± 60.3 (91.2–1,701.4)	12.9 ± 2.9 (2.4–36.9)	6,989.1 ± 459.6 (1,098.0–19,592.7)	7,119.5 ± 409.6 (1,159.7–20,370.4)	1.3 ± 0.4 (0.29–2.5)

ACA, anterior cerebral artery; SD, standard deviation; D, the distance from the peak location to the direct flow impingement center. Peak 1 was located on the arterial branch that formed a narrower angle with the A1 segment, whereas Peak 2 was located on the other arterial branch. ^*^P < 0.05 and ^**^P < 0.01, significantly different between Peak 1 and Peak 2.

For transverse lines, line 4 at the direct flow impinging center had the greatest total pressure (466.3 ± 6.3 Pa) but the least dynamic pressure (44.9 ± 7.4 Pa), vorticity (4,367.7 ± 378.3 1/S), WSS (9.7 ± 0.9 Pa), and strain rate (5,223.6 ± 357.8 1/S) (Table 3). From transverse line 4 to line 1 or 7, as the blood flow was accelerated, the total pressure quickly decreased to 61.9 ± 3.0 Pa on line 1 and 172.3 ± 4.1 Pa on line 7, but WSS, dynamic pressure, vorticity, and strain rate quickly increased to 55.9 ± 2.0 Pa, 6,203.0 ± 176.7 1/S, 10.8 ± 0.1 Pa, and 5,969.6 ± 174.4 1/S, respectively, on line 1 and to 53.0 ± 5.3 Pa, 5,367.4 ± 307.4 1/S, 10.3 ± 0.6 Pa and 5,273.2 ± 286.3 1/S, respectively, on line 7. A significant (*P* < 0.001) difference was found between lines 3 and 5 in terms of dynamic pressure (189.6 ± 4.8 Pa vs. 134.8 ± 6.0 Pa), total pressure (311.5 ± 4.7 Pa vs. 386.0 ± 6.4 Pa), vorticity (10,091.4 ± 571.6 1/S vs. 7,446.5 ± 284.2 1/S), WSS (24.6 ± 0.5 Pa vs. 18.4 ± 0.4 Pa), and strain rate (10,286.2 ± 525.2 1/S vs. 8,022.5 ± 273.0 1/S), with the total pressure significantly greater on line 5 than on line 3 but the dynamic pressure, WSS, vorticity, and strain rate all significantly smaller on line 5 than on line 3. Significant differences were observed between lines 2 and 6 or between lines 1 and 7 (*P* < 0.001, and *P* < 0.01, respectively). Moreover, the dynamic and total pressure, WSS, vorticity, and strain rate on line 3 (189.6 ± 4.8 Pa, 311.5 ± 4.7 Pa, 10,091.4 ± 571.6 1/S, 24.6 ± 0.5, and 10,286.2 ± 525.2 1/S, respectively) were all significantly greater than those on line 2 (87.4 ± 4.0 Pa, 139.5 ± 6.1 Pa, 7,193.5 ± 355.8 1/S, 13.8 ± 0.1 Pa, and 7,256.5 ± 355.3 1/S, respectively) (*P* < 0.001) (Table 3).

**Table 3:** Transverse line hemodynamic stresses at artery bifurcation after virtual aneurysm removal (mean ± SD).

**Transverse line**	**Dynamic pressure (Pa)**	**Total pressure (Pa)**	**Vorticity magnitude (1/S)**	**WSS (Pa)**	**Strain rate (1/S)**
1	158.6 ± 3.5^**^ (132.7–177.4)	210.8 ± 5.2^**^ (162.9–236.1)	10,217.1 ± 927.8^*^ (6,091.3–18,998.4)	20.9 ± 0.6 (17.4–25.3)	10,083.0 ± 871.2^*^ (6,224.9–18,316.3)
2	194.2 ± 5.3^**^ (156.3–217.6)	324.4 ± 5.4^**^ (283.5–351.9)	10,323.69 ± 698.0^**^ (6,845.5–16,313.4)	24.4 ± 0.7^**^ (21.0–28.5)	10,519 ± 631.4^**^ (7,350.1–16,006.2)
3	73.5 ± 2.8^**^ (48.3–90.0)	432.0 ± 3.9^**^ (395.5–451.2)	6,858.6 ± 387.8^*^ (4,928.8–11,080.4)	15.2 ± 0.2 (13.7–17.6)	7,811.9 ± 360.3^*^ (6,045.4–11,864.4)
4	43.7 ± 7.3^#^ (5.6–100.8)	465.3 ± 6.1^#^ (417.9–505.2)	4,254.9 ± 341.0^#^ (2,116.5–7,615.7)	9.5 ± 0.9^#^ (4.3–15.2)	5,434.7 ± 320.8^#^ (3,481.2–8,209.8)
5	105.2 ± 6.1 (73.2–135.9)	456.0 ± 5.0 (422.1–476.6)	7,078.8 ± 255.7 (5,924.9–9,913.2)	15.0 ± 0.3 (13.2–16.9)	7,686.7 ± 267.1 (6,291.4–10,331.5)
6	133.6 ± 7.3 (92.2–166.8)	398.2 ± 7.4 (340.4–420.9)	6,942.7 ± 428.8 (4,830.4–9,453.1)	17.5 ± 0.6 (14.0–20.3)	7,605.3 ± 414.0 (5,576.8–10,014.3)
7	115.4 ± 8.2 (66.8–153.8)	291.5 ± 7.6 (244.2–329.5)	9,410.0 ± 358.4 (7,210.9–11,432.4)	19.7 ± 0.8 (15.1–23.0)	9,623.3 ± 241.7 (7,423.4–11,476.6)

SD, standard deviation. The direct flow impinging center is on line 4. ^*^P < 0.05 and ^**^P < 0.01, significantly different between lines 1 and 7, lines 2 and 6, and lines 3 and 5. ^#^*P* < 0.05 or 0.01, significantly different between line 4 and line 3 or line 5.

### Flow

The blood flow was stagnant at the direct flow impingement center on the bifurcation wall but quickly spread from the center to the bilateral arterial branches. The distance from the direct blood flow impingement on the bifurcation apex (the impingement center) to the location where the flow was parallel (laminar) to the bilateral daughter arterial wall was termed D, which indicated the acceleration area. Ds was from the direct impingement center to the point the flow became laminar on the branching artery that formed a smaller angle with the parent artery, and Di was from the direct impinging center to where the flow became laminar on the other arterial branch that formed a larger angle with the parent artery (Figure 2D). Blood flow needed a longer distance to become laminar in the arterial branch that formed a smaller angle than the other branch that formed a greater angle with the parent artery (Ds > Di). Moreover, in the area immediately adjacent to the impingement center, direction-changed flow produced vortices that were significantly (*P* < 0.001) greater in the arterial branch that formed a smaller angle with the parent artery than the other arterial branch (10,091.4 ± 571.6 1/S on lines 3 vs. 7,446.5 ± 284.2 1/S on line 5, Table 3).

### Bifurcation angles and hemodynamic stresses

**Figure 4:**
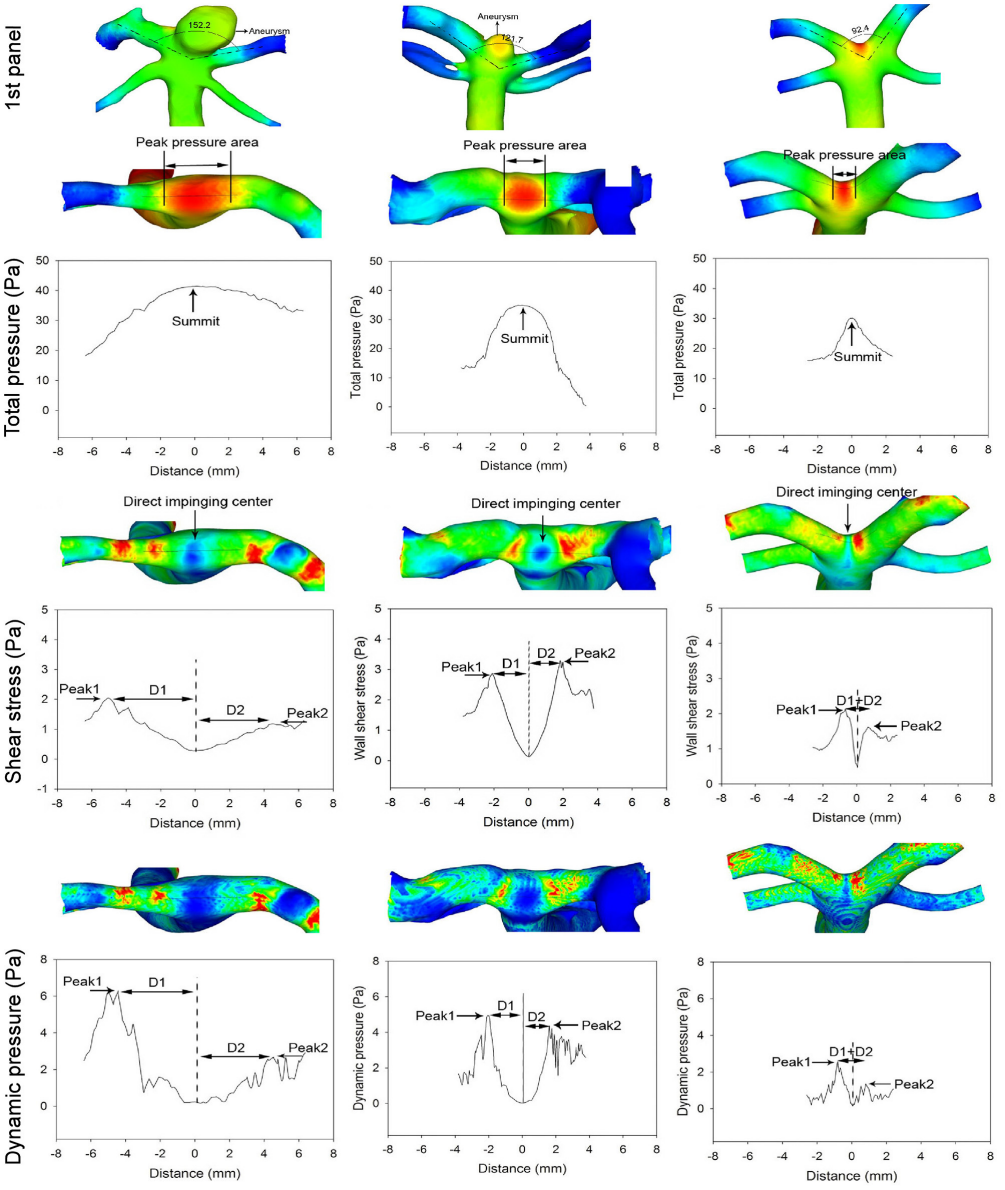
The first panel shows three patients with different basilar artery (BA) bifurcation angles from 152.2° (with a BA aneurysm) to 121.7° (with a BA aneurysm) and 92.4° (with no cerebral aneurysms). The arrow indicates a BA aneurysm. Below the first panel, the top row in the total pressure, shear stress, and dynamic pressure panels shows the hemodynamic stresses on the BA bifurcations after virtual BA aneurysm removal. As the bifurcation angle becomes narrower, the total pressure profile is decreased and narrowed to focus on the bifurcation apex (total pressure panel). The maximal total pressure at the direct flow impinging center decreases from 40.3 to 34.8 Pa and 30.1 Pa, respectively, from 152.2° to 121.7° and 92.4°. Double arrows indicate the total pressure peak region. The shear stress and dynamic pressure panels reveal that with the reduction of the bifurcation angle, both the shear stress and dynamic pressure peaks decrease along with a decrease in the distance between the two peaks. The distance (D1 + D2) between the two peaks is 8.93, 3.6, and 1.6 mm, respectively, in different bifurcations. The arrow in the shear stress panel indicates the direct flow impingement center.

**Figure 5:**
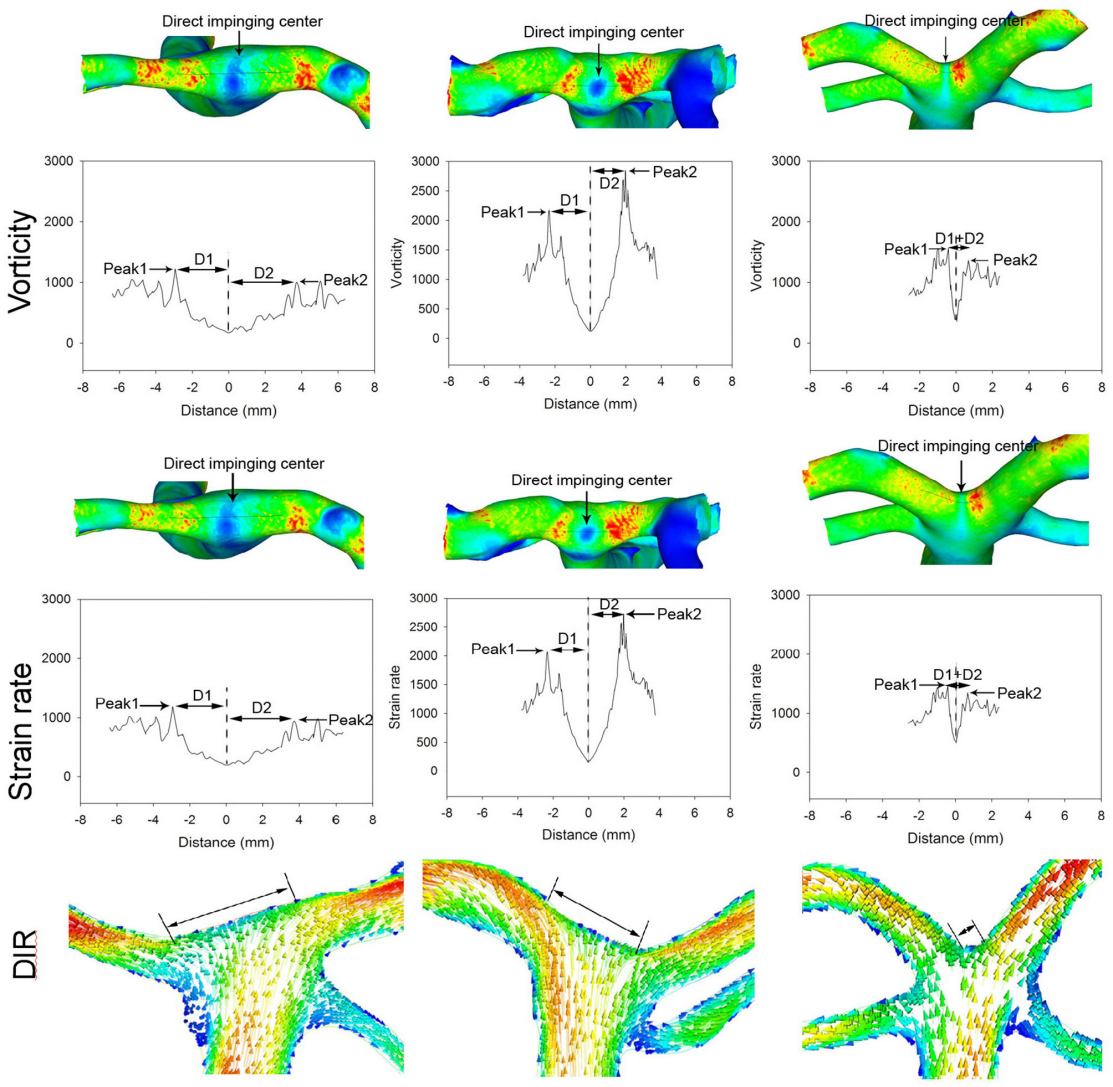
Different basilar artery (BA) bifurcation angles induce varied distributions of strain rate and vorticity at the bifurcation apex (the patient is the same as in Figure 4). With the reduction of the BA bifurcation angle, the peaks of vorticity and strain rate decrease along with a decrease in the distance between the two peaks. The distance (D1 + D2) between the two peaks is 6.7, 4.2, and 0.9 mm, respectively, in different bifurcations. The arrow indicates the direct flow impingement center. The direct flow impingement region (DIR, double arrows) decreases with the reduction in the BA bifurcation angle.

The analysis of patients with different BA bifurcation angles (152.2° with a BA aneurysm, 121.7° with a BA aneurysm, and 92.4° without cerebral aneurysms) after virtual aneurysm removal demonstrated that, with the narrowing of bifurcation angles, all the hemodynamic parameters decreased, the profiles of the parameters became narrowed, and the direct flow impingement region shrank, with the hemodynamic stresses focusing more on the bifurcation wall (Figures 4, 5). As the bifurcation angle dropped from 152.2° to 121.7° and 92.4°, the greatest total pressure at the direct flow impingement center decreased from 40.3 to 34.8 Pa and 30.1 Pa, respectively, and the distance (D1 + D2) between the two stress peaks was reduced from 8.93 to 3.6 mm and 1.6 mm in both WSS and dynamic pressure and from 6.7 m to 4.2 mm and 0.9 mm in both vorticity and strain rate.

## Discussion

The risk factors for intracranial aneurysm formation, including age, gender, hypertension, and hemodynamic stresses, all contribute to the initiation and growth of intracranial aneurysms (14, 15), and hemodynamic stresses are the initial contributing factor for intracranial aneurysms (16). Intracranial aneurysms have been well-observed to occur at intracranial arterial bifurcations that are impinged by excessive hemodynamic pressures (16). In this study, we investigated the hemodynamic forces on the big arterial bifurcation apex, using seven transverse lines and one longitudinal line. We attempted to reveal the exact location of the aneurysm growth. The longitudinal line on the arterial bifurcation wall was to delineate the hemodynamic stress profile experienced by the bifurcation apex, demonstrating one direct impinging center of flow right at the bifurcation wall and two hemodynamic stress peaks on both sides of the impinging center on two branches (Figures 2–4). Seven transverse lines were also used to detect the area on the bifurcation wall that endured stronger hemodynamic stresses (Figure 2; Table 3) for possible aneurysm initiation. Transverse line 4 was localized at the center of the direct flow impact, line 3 was localized at Peak 1 on the arterial branch that formed a smaller angle with the parent artery, and line 5 was localized at Peak 2 on the other arterial branch that formed a greater angle with the parent artery. The area around line 3 at Peak 1 experienced significantly greater hemodynamic stresses, except for total pressure, than line 5 at Peak 2, indicating that the area around line 3 at Peak 1 on the arterial branch that formed a smaller angle with the parent artery might be more easily affected by abnormally enhanced hemodynamic stresses for aneurysm development.

Our study found that the arterial bifurcation angle with a bifurcation aneurysm was significantly larger than that of non-aneurysm bifurcations (*P* < 0.001), whereas the angle between the arterial branch and the parent artery in patients with bifurcation aneurysms was significantly (*P* < 0.05) smaller than that in patients without aneurysms. Our findings were similar to one study (17) that reported that the smaller angle formed between the parent A1 and A2 segment was significantly associated with ACA bifurcation (Acom) aneurysms and that Acom aneurysms are more common at larger than at smaller arterial bifurcations. In our study, most (68/80) of the bifurcation aneurysms deviated to the arterial branch that formed a smaller angle with the parent artery (Figures 1, 2). This may suggest that the direct flow impingement center with the flow perpendicular to the bifurcation apex was not the initiating aneurysm site, and the Peak 1 location with the maximal hemodynamic stresses on the arterial branch that formed a smaller angle with the parent artery might be more significantly vulnerable to hemodynamic stress damage for aneurysm development. From the direct flow impingement center to the hemodynamic stress peak was the flow-accelerating area, and as the flow was quickly accelerated, the hemodynamic stresses were increasingly enhanced to reach the peak values before the flow became laminar. The dynamic pressure, vorticity, WSS, and strain rate at Peak1 on the arterial branch that formed a smaller angle with the parent artery were significantly (*P* < 0.05) increased compared with those at Peak 2 on the arterial branch that formed a larger angle with the parent artery. This may indicate that, on entering the arterial branch that formed a narrower angle with the parent artery, the blood flow greatly enhanced the hemodynamic stresses to damage the vessel wall at the peak site of the stresses for inducing an aneurysm. At the direct flow impingement center where the flow direction was perpendicular to the bifurcation wall, the total pressure reached the maximum, whereas the dynamic pressure, WSS, vorticity, and strain rate dropped to their lowest points. Low WSS only enhanced endothelial hyperplasia (18) and vascular intimal thickening (19) rather than destroying the arterial wall (which may initiate an aneurysm). Furthermore, the distance was significantly longer from the direct flow impingement center to Peak 1 than to Peak 2, suggesting that the wall at Peak 1, which experiences greater hemodynamic stresses, is more vulnerable to aneurysm development and requires a longer distance for blood flow to resume to a laminar state (Table 3).

Laminar flow at straight arterial segments produces WSS with a function of endothelial cell protection, whereas WSS at the flow-accelerating areas on both sides of the direct flow impinging center induces endothelial damage and enables the vascular wall to become susceptible to destructive remodeling for aneurysm formation (5, 20, 21). This may suggest that WSS plays a vital role in aneurysm initiation. In this study, it was found that most of the bifurcation aneurysms deviated to the arterial branch that formed a narrower angle with the parent artery and that the peak hemodynamics stresses, including WSS, vorticity, dynamic pressure, and strain rate, were all significantly larger on the arterial branch that formed a smaller angle than on the other branch that formed a larger angle with the parent artery (*P* < 0.001) (Tables 2, 3). This finding may indicate that, besides WSS, these hemodynamic stresses also play a vital role in inducing bifurcation aneurysms.

In the analysis of the hemodynamic stresses sampled by some lines on the bifurcation apex, transverse lines 1, 5, 6, and 7 were localized on the bifurcation wall outside the aneurysm, implying that these regions with their specific hemodynamic forces are not the original aneurysm-triggering location. Transverse lines 2–4, which were localized within the aneurysm range, were probably the aneurysm-triggering location. Line 4 was positioned at the direct flow impingement center and had the maximal total pressure but minimal dynamic pressure, vorticity, WSS, and strain rate. Lower WSS can only promote arterial intimal thickening rather than destructive remodeling (19) and, thus, line 4 cannot be the aneurysm-triggering location. The mean total and dynamic pressure, vorticity, strain rate, and WSS on line 3 at Peak 1 were significantly increased (*P* < 0.001) compared with those on line 2, which probably suggests that the hemodynamic forces depicted by line 3 at Peak 1 on the arterial branch that formed a narrower angle with the parent artery are significant enough to trigger an aneurysm (Figure 3; Table 2). This may imply that only when both WSS and pressure reach a certain degree, can an intracranial aneurysm be triggered.

Arterial bifurcation angles can affect the development of flow turbulences at the arterial bifurcation (22). In this study, we also investigated the possible relationship between blood flow direction, flow velocity, and bifurcation aneurysm development on the major arterial bifurcation. In the parent artery, the blood flow with a greater velocity moved toward and directly impacted the bifurcation wall, causing additional pressure (besides static blood pressure). This additional pressure was transformed from the kinetic energy of the moving blood flow upon hitting the bifurcation wall, leading to maximal pressure on the bifurcation wall. Then, the disturbed flow continued to move toward the bilateral arterial branches. In the arterial branches, the disturbed flow was quickly sped up and returned to the laminar status again. When the blood flow was accelerated, WSS also increased quickly to reach the peak value and impair the endothelial cell and vascular wall, possibly triggering an aneurysm. From the disturbed condition at the direct flow impingement center to the laminar condition at both arterial branches, the blood flow took a longer path in the arterial branch that formed a narrower angle than the other branch that formed a wider angle with the parent artery, which may suggest that the vascular branch that formed a narrower angle with the parent artery may be more vulnerable to destructive remodeling to trigger an aneurysm.

In the analysis of the hemodynamic forces on the major artery bifurcations before and after virtual aneurysm removal, the hemodynamic forces on the aneurysm dome were significantly (*P* < 0.001) lower than those at the aneurysm-triggering location. In the analysis of line-sampled hemodynamic stresses, the total pressure, dynamic pressure, strain rate, vorticity, and WSS on the aneurysm dome were very low, which suggests that aneurysm development involves the release of the local abnormally increased hemodynamic forces at the aneurysm-triggering location (Figure 3). The WSS is greater at the peak of hemodynamic stresses corresponding to the aneurysm-triggering location and can promote the production of the matrix metalloproteinases (MMPs) (23), and MMPs are able to degrade the elastin in the arterial extracellular matrix, resulting in the internal elastic lamina destruction and medial thinning (24). The resultant thinned vascular wall in the peak area adjacent to the direct flow impingement center will protrude outward under high pressures to reduce the abnormally increased WSS to a physiological degree. This may be due to the mechanism of aneurysm initiation. In this study, we first investigated the hemodynamic forces on the aneurysm dome and at the aneurysm-triggering location after virtual aneurysm removal, and the total pressure, dynamic pressure, strain rate, WSS, and vorticity were all significantly increased at the aneurysm-triggering location compared with those on the aneurysm dome (*P* < 0.001). This suggests that vascular protrusion and aneurysm development involve the release of high local hemodynamic forces, including total pressure, dynamic pressures, and WSS.

In our study, the arterial bifurcation angle at all four major cerebral artery bifurcations had a significant (*P* < 0.001) positive correlation with the patient's age, indicating that the arterial bifurcation angle increases with age. Moreover, the arterial bifurcation angle was significantly (*P* < 0.001) wider in patients with aneurysms than those without bifurcation aneurysms. Cerebral aneurysms tend to occur in patients at ~50 years of age (25–27). With age, the arterial bifurcation angle also increases, which is accompanied by an increase in peak hemodynamic stresses and their distance (D1 + D2) on the bifurcation apex, causing destructive wall remodeling and leading to possible aneurysm formation. The distance (D1 + D2) between the two hemodynamic stress peaks reflected the region of action by these maximal hemodynamic stresses on the bifurcation wall. The shorter the distance, the smaller the region of action by these stresses and the more focused the hemodynamic forces. The alteration of the arterial bifurcation angle is accompanied by an increase or decrease in the peak hemodynamic stresses and their distance (D1 + D2) on the bifurcation apex. Because the arterial bifurcation wall is protected from hemodynamic forces by a narrowed band of dense collagen fibers that cover the bifurcation apex to provide strength and stiffness to this region (28–30), an increase in the peak distance (D1 + D2) corresponding to a widened bifurcation angle may extend the peak hemodynamic forces beyond the protection of the densely packed collagen fiber band, leading to possible destructive remodeling and aneurysm formation on the adjacent vessel wall by the peak hemodynamic stresses. Contrarily, the narrowing of the arterial bifurcation angle will bring about decreases in both the peak hemodynamic stresses and their distance (D1 + D2), resulting in a smaller region of action by the peak hemodynamic stresses within the protection of the dense collagen fiber band. In our study, when the distance (D1 + D2) between the peak hemodynamic stresses decreased from 8.93 or 6.7 mm in the 152.2° bifurcation to 1.6 or 0.9 mm in the 92.4° bifurcation angle, the peak hemodynamic stresses decreased and became more focused on the bifurcation apex, a narrow area that is protected by the densely packed collagen fiber band, probably decreasing the possibility of aneurysm initiation.

Aneurysms on the ACA bifurcation (or Acom aneurysms) account for over 25% of all intracranial aneurysms (8). The ACA bifurcation is at the junction of the A2 and the Acom. The Acom communicates between the left and right ACA at the junction of A1–A2. Prior to the Acom, the artery is A1, and post the Acom, the artery becomes the A2 segment. Most of the time, the Acom is closed. Even without blood flow through the Acom, blood flow from the A1 segment directly impacts the A2 segment junction and the Acom (the junction is the aneurysm initiation site), increases the hemodynamic stresses on the junction, and induces aneurysm development. At the time of aneurysm initiation, blood is not needed to flow through the Acom to the other side. When blood flows at a lower pressure on one side (left or right), the Acom is opened and provides blood flow to the other side at a lower pressure, and in this case, the blood passes through the Acom to increase the area of direct flow impact on the junction and promote hemodynamic stresses and subsequent aneurysm formation. Enlarged ACA bifurcation angles (the angle formed between the A2 and Acmm) enhance hemodynamic stresses to initiate aneurysm development at the A2 and Acom junctions (31, 32). This is why Acom aneurysms are so frequent and account for over 25% of all intracranial aneurysms (8). In the clinical setting of treating cerebral aneurysms endovascularly, stents have been used to help assist coiling embolization, and the deployment of stents at the arterial bifurcation has been found to efficiently decrease the bifurcation angle and, consequently, abnormal hemodynamic stresses (10, 33), thus protecting the arterial bifurcation wall from damage, destructive remodeling, and subsequent aneurysm formation or recurrence. Intracranial stents have been used to assist the embolization of intracranial aneurysms at all four big arterial bifurcations and contribute to eliminating pathogenic factors of aneurysm initiation at these locations.

Our study, different from other studies (34–36), investigated the general patterns of flow and hemodynamic stresses in a few models for assessing the risk of intracranial aneurysms. In our study, a large amount of patients' specific three-dimensional imaging datasets were used to study the flow patterns on the arterial bifurcation wall with and without bifurcation aneurysms for delineating the possible mechanisms of aneurysm initiation caused by enhanced hemodynamic stresses. Moreover, we employed the virtual removal technique to remove the aneurysm and restore the artery to the status before the aneurysm was formed. In this way, we tried to delineate the exact location of aneurysm initiation caused by abnormally increased hemodynamic stresses on the bifurcation wall.

This study had some limitations. In the blood flow simulation for CFD analysis, we used the flow inlet parameters of 0.1 m/s for velocity and 0 for pressure at the outlet for simulation in the intracranial arteries. If the inlet was at the beginning of ICA, internal carotid artery; BA, basilar artery, this inflow value of 0.1 m/s may not be sufficient for reflecting the actual value. However, the CFD study aimed to reach statistically significant values and differences, and in this aspect, we did reach a significant difference in patients with and without bifurcation aneurysms or in patients with narrowed or widened bifurcation angles. Moreover, real arteries are elastic and pulsate during systole, and in our study, we used rigid non-slip walls for CFD simulation, which is also a relative limitation when interpreting the data from CFD models.

In summary, the hemodynamic stresses in the acceleration region near the direct flow impingement center at major arterial bifurcations were all increased on the arterial vessel that formed a narrower angle with the parent artery, probably triggering an aneurysm to release the local abnormally increased hemodynamic stresses induced by the direct impingement of blood flow.

## Data availability statement

The raw data supporting the conclusions of this article will be made available by the authors, without undue reservation.

## Ethics statement

Ethical review and approval was not required for the study on human participants in accordance with the local legislation and institutional requirements. Written informed consent from the patients/participants or patients/participants' legal guardian/next of kin was not required to participate in this study in accordance with the national legislation and the institutional requirements.

## Author contributions

HG: Data curation, Formal analysis, Funding acquisition, Investigation, Validation, Visualization, Writing—original draft. J-FL: Data curation, Investigation, Methodology, Supervision, Validation, Visualization, Writing—review and editing. C-HL: Conceptualization, Formal analysis, Investigation, Methodology, Project administration, Supervision, Validation, Writing—review and editing. J-WW: Data curation, Investigation, Methodology, Resources, Validation, Visualization, Writing—review and editing. HL: Data curation, Investigation, Methodology, Resources, Supervision, Validation, Visualization, Writing—review and editing. B-LG: Conceptualization, Data curation, Formal analysis, Investigation, Supervision, Validation, Visualization, Writing—review and editing.
